# The Crosstalk between Gut Microbiota and Bile Acids Promotes the Development of Non-Alcoholic Fatty Liver Disease

**DOI:** 10.3390/microorganisms11082059

**Published:** 2023-08-11

**Authors:** Zhonglin Li, Hang Yuan, Huikuan Chu, Ling Yang

**Affiliations:** Division of Gastroenterology, Union Hospital, Tongji Medical College, Huazhong University of Science and Technology, 1277 Jiefang Avenue, Wuhan 430022, China; d202081716@hust.edu.cn (Z.L.); m202175811@hust.edu.cn (H.Y.); 2012xh0827@hust.edu.cn (H.C.)

**Keywords:** bile acid, bile acid receptor, gut microbiota, NAFLD, NAFLD treatment

## Abstract

Recently the roles of gut microbiota are highly regarded in the pathogenesis of nonalcoholic fatty liver disease (NAFLD). The intestinal bacteria regulate the metabolism of bile acids depending on bile salt hydrolase (BSH), 7-dehydroxylation, hydroxysteroid dehydrogenase (HSDH), or amide conjugation reaction, thus exerting effects on NAFLD development through bile acid receptors such as farnesoid X receptor (FXR), Takeda G-protein-coupled bile acid protein 5 (TGR5), and vitamin D receptor (VDR), which modulate nutrient metabolism and insulin sensitivity via interacting with downstream molecules. Reversely, the composition of gut microbiota is also affected by the level of bile acids in turn. We summarize the mutual regulation between the specific bacteria and bile acids in NAFLD and the latest clinical research based on microbiota and bile acids, which facilitate the development of novel treatment modalities in NAFLD.

## 1. Introduction

NAFLD has become the most common chronic liver disease in both Western countries and China [1,2], and patients with NAFLD have a high risk of liver-related mortality along with metabolic comorbidities and extra-hepatic diseases. The pathogenesis of NAFLD includes insulin resistance in adipose tissue, abnormal de novo lipogenesis, the influence of lipotoxic lipids, and the evolution of the intestinal microbiome [3]. The significantly altered microbiota signature in NAFLD is increased *Proteobacteria* (in phylum); increased *Enterobacteriaceae* and decreased *Ruminococcaceae* and *Rikenellaceae* (in family); and increased *Dorea* and decreased *Faecalibacterium*, *Coprococcus*, and *Anaerosporobacter* (in genus) [4]. Cohousing experiments with mice or fecal transplant experiments have provided evidence of a determinant role of gut microbiota in NAFLD development. The mechanism of how bacterial communities regulate the development of NAFLD is mainly attributed to the change of gut-derived metabolites such as lipopolysaccharide, short-chain fatty acids (SCFAs), and bile acids. However, there is still limited knowledge about the microbial regulation of bile acids in the scene of NAFLD, because of the great diversity of microbes and the limited ways to identify their influence on the underlying pathophysiologic mechanism.

NAFLD patients have increased bile acids and the total cholate to chenodeoxycholate ratio. The NAFLD activity score is positively correlated with conjugated cholate and taurocholate and negatively correlated with the secondary to primary bile acid ratio [5]. Bile acids are ligands of the nuclear receptors FXR, VDR, and pregnane X receptor (PXR) as well as cell surface G-protein-coupled receptors (GPCR) such as TGR5 and sphingosine-1-phosphate receptor (S1PR)2 [6]. These receptors control the genes involved in carbohydrate and lipid metabolism, energy expenditure, and inflammation response by activating transcriptional networks and signaling cascades in entero-hepatic tissues. Gut microbiota affect bile acid composition and regulate NAFLD development through bile acid receptors. Targeting the bile acid receptors provides a new approach to treating and preventing NAFLD. Using the ligand of FXR named obeticholic acid (OCA) has been proven to improve insulin sensitivity in phase II and III trials in patients with nonalcoholic steatohepatitis (NASH) [7]. Bile acids could also transform the constitution of gut microbiota in return. Thanks to the development of high-throughput sequencing technology, multi-omics analyses have been successfully applied in revealing the interaction of intestinal bacteria and bile acids to help further discover the potential therapeutic targets for NAFLD. 

This article summarizes the specific regulatory relationship between the gut microbes and bile acids reported in the latest years and discusses the underlying mechanism of how the gut microbiota—bile acid pathway influences the development of NAFLD.

## 2. Interaction between Bile Acids and the Gut Microbiota

Bile acid synthesis takes place in the liver and the bile acids are conjugated with glycine or taurine and then actively transported into bile until released into the duodenum after meal ingestion. Approximately 95% of biliary-secreted bile acids are reabsorbed from the intestine by the apical sodium-dependent bile acid transporter (ASBT) and recirculated to the liver via the portal vein, from which they are secreted again, which is called enterohepatic circulation.

The synthesis of bile acids is regulated by gut microbiota. The bile acid level is decreased in the gallbladder and small intestine and increased in the cecum, colon, and feces in the presence of gut microbiota. And bile acids were more chemically diverse in conventionally raised mice than germ-free mice. Gut microbiota suppresses the expression of ASBT to influence the reabsorption of bile acid in the intestine and inhibits bile acid synthesis by removing the FXR antagonists taurine-α-muricholic acid (TαMCA) and TβMCA [8].

Gut microbiota has participated in the process of biotransformation and modification of bile acids (Table 1). The intestinal bacteria could catalyze bile acid deconjugation to prevent bile acid active reuptake from the small intestine. It depends on bacteria with BSH activity, which is present in all major bacterial divisions including *Firmicutes*, *Bacteroidetes*, and *Actinobacteria* [9]. Deconjugated primary bile acids that escape uptake then enter into the colon, where they are metabolized into secondary bile acids through 7-dehydroxylation, that is transforming chenodeoxycholic acid (CDCA) into lithocholic acid (LCA) and transforming cholic acid (CA) into deoxycholic acid (DCA). This process solely depends on a few species of anaerobic gut bacteria in the genus *Clostridium* [9]. In humans, CDCA also can be transformed into ursodeoxycholic acid (UDCA) by *Clostridium absonum* [10]. In mice, secondary bile acids include DCA, LCA, ωMCA, hyodeoxycholic (HDCA), and murideoxycholic acid (MDCA). The hydroxy groups of bile acids can be oxidized and epimerized by microbiota that have HSDH enzymes such as *Actinobacteria*, *Proteobacteria*, *Firmicutes*, and *Bacteroidetes* [11].

In addition to the classic microbial metabolism of bile acids, a completely different mechanism of microbiome-mediated amide conjugation of the cholate backbone with the amino acids phenylalanine, tyrosine, and leucine has been discovered recently. The novel bile acids named phenylalanocholic acid (Phe-chol), tyrosocholic acid (Tyr-chol), and leucocholic acid (Leu-chol) are found to exist in both mice and human intestines, and Phe-chol and Leu-chol are more abundant in mice fed a high-fat diet (HFD) and patients with fatty guts. *Clostridia bolteae* strain WAL-14578 and strain CC43001B could synthesize both Phe-chol and Tyr-chol [12].

In reverse, the composition of bile acids also has been shown to regulate the germination of intestinal flora. It is reported that secondary bile acids could suppress *Clostridium difficile* germination, which is protective against infection by *Clostridium difficile* [10]. The composition of the gut bile acid pool modulates the composition of gut flora by regulating colonic FOXP3+ regulatory T cells [13]. Fxr-deficient mice have bile acid composition alteration, resulting in the increase in *Firmicutes* and the decrease in *Bacteroidetes* [14]. Using the analog of the gut hormone fibroblast growth factor (FGF)19 named aldafermin decreases the levels of glycocholic acid (GCA), glycochenodeoxycholic acid (GCDCA), glycodeoxycholic acid (GDCA), and DCA in patients with NASH. These bile acids might be toxic to *Veillonella* as it is increased after administration of aldafermin, which enhances NASH patient performance by degrading lactate to produce the SCFA propionate [15]. Another example is the alteration of gut flora in primary biliary cholangitis and its restoration after UDCA therapy [16].

## 3. The Alteration of Gut Flora along with Bile Acids in NAFLD and Metabolic Disorders

The feature of the NAFLD serum bile acid profile is the elevated bile acid production and the increase in the FXR antagonistic DCA with the decrease in the agonistic CDCA [17]. Several bile acids derived from DCA such as glycodeoxycholate (GDCA), 7-ketodeoxycholic acid, and dehydrocholic acid also increase with NAFLD activity and fibrosis stage. The synthesis of DCA and downstream metabolite requires the increased expression of microbial BSH, bile acid operon (BaiCD), and HSDH. *Bacteroidetes* and several genera of the *Lachnospiraceae* family containing DCA-generating genes increase with increasing disease severity, whereas *Ruminococcaceae* is decreased because it is sensitive to the antibacterial effects of DCA [18].

A systemic summary of the interactions between gut microbiota and bile acids in NAFLD could provide clues about their mechanism of action (Table 2). It is proven that *Parabacteroides distasonis* could ameliorate weight regain by elevating levels of the non-12α-hydroxylated bile acids, LCA, and UDCA in mice [19]. Administration of vancomycin in male obese subjects aggravates insulin resistance by decreasing *Firmicutes* and increasing *Proteobacteria*, followed by a marked reduction in secondary bile acids [20]. The reduction of *Ruminococcaceae_NK4A214_group*, which has a relationship with the decrease in CA, LCA, and tauroursodeoxycholic acid (TUDCA), disturbs the absorption of vitamin A in male infertility in a metabolic syndrome sheep model [21]. It is reported that eicosapentaenoic acid (EPA) and docosahexaenoic acid (DHA) can prevent the development of metabolic disorders. KEGG analyses of the gut microbiome and metabolome consistently demonstrated that the key metabolic pathway modified by DHA/EPA is the bile acid metabolism pathway. DHA/EPA-fed mice have reduced fecal levels of TCA and taurochenodeoxycholic acid (TCDCA) and increased levels of CA and CDCA because DHA/EPA could enrich microbial genes encoding BSH. This study also indicates that the abundance of *Barnesiella* (OTU136) is inversely associated with taurine-conjugated bile acids (TCA  +  TCDCA) while *Clostridium XlVa* (OTU57) is positively related to fecal DCA level. The changed bile acids downregulate hepatic gluconeogenesis through activating FXR-SHP-FOXO1 signaling [22,23]. In another experiment using oligofructose-enriched inulin to treat children obesity, oligofructose-enriched inulin causes significant increases in species of the genus *Bifidobacterium* and decreases in *Bacteroides vulgatus*, inducing the increase in primary bile acids in feces [24].

## 4. The Mechanism of How Gut Microbiota Influences the Progression of NAFLD through Bile Acids

### 4.1. FXR

#### 4.1.1. Gut Microbiota Have an Impact on NAFLD through Bile Acid–FXR Pathway

FXR is highly expressed in the liver and ileum. In the liver, bile acid-activated FXR inhibits the expression of cholesterol 7-alpha- hydroxylase (CYP7A1) via the induction of small heterodimer partner (SHP; NR0B2). CYP7A1 is generally considered the rate-limiting enzyme that initiates bile acid synthesis. The activation of FXR in the distal ileum induces the expression of Fgf15 (FGF19 in humans), which binds to the FGF receptor 4 (FGFR4)/β-klotho heterodimer complex when it reaches the liver by portal blood to inhibit CYP7A1 expression [38]. FGF15/19 could also inhibit bile acid generation by increasing the stability of SHP [39]. Bile acid affinities for FXR are as follows: CDCA > LCA = DCA > CA [40]. Phe-chol and Tyr-chol are also strong human FXR agonists. It is proven that Phe-chol is an agonist twice as strong as CDCA. These bile acids could increase the expression of the FXR effector genes Fgf15 in the intestine and Shp in both the intestine and liver [12].

Research about cholesterol gallstone disease finds that the bacterium *Desulfovibrionales* is associated with enhanced cecal secondary bile acid production and is enriched in patients with gallstone disease. H_2_S is the product of *Desulfovibrionales* that could induce hepatic FXR and inhibit CYP7A1 expression, which results in the accumulation of cholesterol in the liver [25]. Activating hepatic FXR could ameliorate NASH according to research about the application of disulfiram in the treatment of NASH. Disulfiram inhibits the growth of *Clostridium* and reduces *Clostridium*-mediated 7α-dehydroxylation activity to suppress secondary bile acid biosynthesis, which in turn activates hepatic FXR signaling to ameliorate NASH [26]. Hepatocyte MyD88 ablation exhibits an increase in *Ruminococcus* and *Oscillospira* and a decrease in *Sutterella* and *Allobaculum*, resulting in the decrease in the main FXR agonist CA and the increase in FXR antagonist T-βMCA, thereby contributing to glucose intolerance, inflammation, and hepatic insulin resistance in mice [27].

Interestingly, activation of the FXR in the intestine seems to have the opposite effect on NAFLD. Intestine-specific Fxr disruption ameliorates hepatic triglyceride accumulation in mice under a high-fat diet [41]. Theabrownin reduces BSH-enriched bacteria and increases the levels of ileal conjugated bile acids to inhibit the intestinal FXR-FGF15 signaling pathway, resulting in increased hepatic production and fecal excretion of bile acids, which in turn reduces hepatic cholesterol and decreases lipogenesis [28]. Type 2 diabetes (T2D) patients treated with metformin revealed a decrease in *Bacteroides fragilis* and an increase in FXR antagonist glycoursodeoxycholic acid (GUDCA), which supports the beneficial effect of intestinal FXR antagonist to metabolic dysfunction [29]. The Western diet induces an increase in the abundance of *Firmicutes* and a relative reduction in the abundance of *Bacteroides*, which mediates significantly increased DCA and LCA. DCA-mediated FXR activation in the myeloid cells in the intestine can lead to the production and activation of type I IFN resulting in the dysfunction of intestinal Paneth cells and intensifying gut inflammation in mice [30]. Another study has proven that hepatic thyroid hormone signaling modulates glucose homeostasis through repressing intestinal FXR signaling [42].

However, there are opposing conclusions concerning the effect of intestinal FXR agonists.

*Lactobacillus rhamnosus GG-*treated mice showed a reduction in taurine-β-muricholic acid (T-βMCA), an FXR antagonist, and the amelioration of liver inflammation and fibrosis, while these changes are reversed by intestine-specific FXR inhibitors. It proves that increasing the intestinal FXR signaling pathway leads to the suppression of bile acid de novo synthesis and prevents excessive bile acid-induced liver injury and fibrosis in mice [31]. And both liver and intestine FXR agonists could reduce bacterial translocation via the portal-venous route to the liver in cirrhosis [43]. Application of the intestine-restricted FXR agonist fexaramine (FEX) markedly increases taurolithocholic acid (TLCA) and improves metabolism indicators in *db/db* mice. FEX increases the transformation of LCA from CDCA by amplifying the amount of *Acetatifactor* and *Bacteroides*, which have high BSH and 7α- and 7β-dehydroxylase activity. LCA then activates TGR5 signaling to stimulate GLP-1 secretion from L cells [44], resulting in promoting adipose tissue browning, improving hepatic insulin signaling and glucose metabolism [32]. Another research study found increased levels of total and taurine-conjugated bile acid pool sizes and intestinal FXR signaling after Roux-en-Y gastric bypass (RYGB) surgery, which is linked to increased TGR5 expression and stimulates adaptive thermogenesis in rats [45]. As there is a frequent substance exchange between the gut and brain, it is intriguing to find that inhibition of FXR in the dorsal vagal complex enhances insulin action in HFD-fed rats [46].

**Figure 1:**
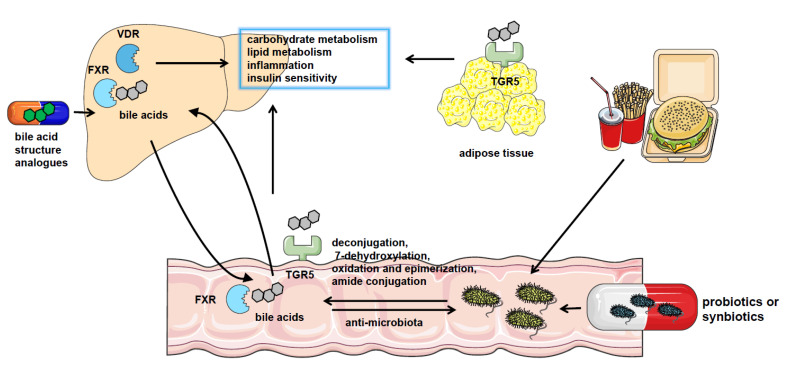
The crosstalk between gut microbiota and bile acids in NAFLD. NAFLD patients have significantly altered gut microbiota composition and bile acid pool. Gut microbiota catalyzes bile acid metabolism via deconjugation, 7-dehydroxylation, oxidation, and epimerization or amide conjugation. The bile acids could also regulate the constituents of the gut microbiome by the detergent actions. Bile acids play a role in regulating nutrient metabolism in NAFLD by binding with bile acid receptors such as FXR, VDR, and TGR5 in the liver, intestine, and adipocytes or other tissues. And pharmacological treatment using probiotics or synbiotics or applying analogs of bile acids are effective approaches to the therapy of NAFLD.

In conclusion, gut microbiota transforms bile acid composition to regulate FXR distributed in the different locations and influences the metabolism status in NAFLD as shown in Figure 1.

#### 4.1.2. The Molecular Mechanism of How FXR Influences NAFLD

The expression of FXR is suppressed by iron [47]. FXR transcription activation could also be regulated by glucose via FXR own protein O-GlcNAcylation [48]. The histone deacetylase Sirtuin (SIRT) overexpression causes deacetylation of FXR with low FXR protein expression. And it could also regulate FXR target gene transcription by influencing histone methylation [49].

In NAFLD, FXR activation induces the expression of transporters that provide outflow routes for bile acid efflux to avoid toxic bile acid overload, such as bile acid export pump (BSEP) [50] and organic solute transporter-alpha and -beta (OSTα/β). The inhibition of ASBT by FXR in the enterocytes also prevents the uptake of bile acids [51]. It also upregulates the expression of ATP-binding cassette sub-family G member 5/8 (ABCG5/8) that is in charge of cholesterol efflux [52]. Accordingly, intestinal FXR activation renders the bile acid pool more hydrophylic and less efficient in emulsifying lipids, resulting in cholesterol fecal elimination via ABCG5/8 [53].

**Figure 2:**
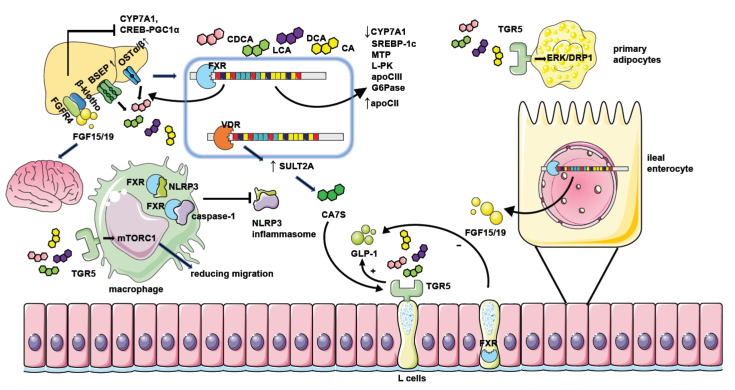
The mechanism of NAFLD regulation by bile acid receptors. Hepatic FXR activation suppresses bile acid synthesis by inhibiting CYP7A1 expression. Intestinal FXR activation could also inhibit CYP7A1 by inducing the generation of FGF15/19 and modulating gene transcription of CYP7A1 through binding with FGFR4/β-klotho receptors in the liver. Hepatic FXR activation promotes bile acid efflux by increasing the expression of bile acid transporters such as BSEP and OSTα/β thus ameliorating the toxic effect of excessive bile acids. FXR activation when binding with bile acids leads to decreased expression of genes involved in lipid or glucose metabolism including SREBP-1c, MTP, L-PK, apoCIII, and G6Pase. It could also repress the CREB-PGC1α pathway by inducing FGF15/19. Together, these factors result in reduced hepatic triglyceride accumulation and improved insulin sensitivity. Moreover, FGF15/19 could also facilitate weight loss by combining with nerve cell receptors. FXR activation in macrophages inhibits the assembling of the NLRP3 inflammasome and ameliorates hepatic inflammation in NAFLD. The activation of TGR5 in L cells causes the secretion of GLP-1 to potentiate insulin secretion, and the activation of TGR5 in primary adipocytes enhances ERK-DRP1 to increase energy expenditure. However, the activation of FXR in L cells inhibits GLP-1 secretion. TGR5 activation in macrophages also suppresses inflammation response by manipulating mTORC1. Hepatic VDR activation increases the generation of CA7S by promoting SULT2A, which stimulates TGR5 in L cells to promote GLP-1 secretion.

FXR activation results in a reduction in sterol regulatory binding protein-1c (SREBP-1c) expression by the FXR-SHP axis and represses hepatic de novo lipogenesis [54]. The increased expression of SHP after FXR activation suppresses hepatocyte nuclear factor 4α (HNF4α) activities. HNF-4 promotes the expression of microsomal triglyceride transfer protein (MTP), which is involved in transferring triglycerides, cholesterol esters, and phospholipids to newly synthesized apolipoprotein (apo) B [55]. FXR could also decrease apolipoprotein CIII (apo CIII) promoter activity [56] and induce the gene expression of apoC-II [57]. It is known that apo CIII inhibits triglyceride lipolysis whereas apo CII is responsible for triglyceride hydrolysis in chylomicrons and very low-density lipoprotein (VLDL). FXR also regulates glucose metabolism via the upregulation of SHP and represses gluconeogenic gene glucose-6-phosphatase (G6Pase) expression [23]. On the other hand, the activation of FXR also represses the expression of the liver-type pyruvate kinase gene (L-PK) through interacting with carbohydrate-responsive element binding protein (ChREBP) and HNF4α to result in the release of these transcription factors from the promoter of L-PK and repressing hepatic glycolysis [58]. In addition, FXR activation protects NAFLD by blunting hepatic inflammation. FXR interacts with NLRP3 and caspase 1, thereafter preventing NLRP3 inflammasome assembly and activation in macrophages [59], which is contradicted with the increased inflammation caused by the elevation of type I IFN after FXR activation in the myeloid cells in the intestine [30] (Figure 2). Moreover, FXR activation in hepatic stellate cells (HSCs) is reported to alleviate liver fibrosis via the inhibition of TGF-β/SMAD3 in a SHP-dependent way [60] or triggering the expression of anti-fibrotic genes, like peroxisomal proliferator-activated receptor γ (PPAR γ) [61].

FGF15/19, the effector of FXR activation, could also regulate metabolism directly. It could inhibit hepatic gluconeogenesis by inhibiting the cAMP regulatory element-binding protein (CREB)-peroxisome proliferator-activated receptor γ coactivator-1α (PGC-1α) pathway [62]. FGF19 treatment prevents the accumulation of lipid droplets in Fxr-null mice [63] and inhibits lipogenic enzyme expression by elevation in SHP expression [64]. Moreover, FGF15/19 could reach the brain and mediate beneficial weight loss [65,66]. But knockdown of the obligate coreceptor mediating FGF15/19 signaling named β-klotho shows resistance to diet-induced obesity in mice [67].

### 4.2. TGR5

#### 4.2.1. Gut Microbiota Have an Impact on NAFLD through Bile Acid-TGR5 Pathway

TGR5 is a cell surface receptor belonging to the GPCR, which is expressed in enteroendocrine L cells, white and brown adipose tissue, skeletal muscle, gallbladder, non-parenchymal liver cells, and the brain [6]. The order of activation of TGR5 by bile acids is LCA > DCA > CDCA > CA [6]. In addition to bile acids, some steroid hormone intermediates, such as pregnandiol, are also the ligands of TGR5 [68].

Oligofructose could reduce body weight gain and improve glucose metabolism in mice fed a Western-style diet. It enriches bacteria belonging to *Lachnospiraceae* and *Eggerthellaceae* families, which are strongly correlated with cecal HDCA levels. HDCA is elevated after oligofructose administration and it activates TGR5 to stimulate GLP-1 secretion to relieve metabolism disorder [33]. Research shows that intestinal hypoxia-inducible factor 2α (HIF-2α) ablation in mice leads to lower lactate levels by controlling the expression of intestinal Ldha, which in turn results in less *Bacteroides vulgatus* and greater *Ruminococcus torques* abundance. Together, these changes elevate taurine-conjugated cholic acid (TCA) and DCA levels and activate TGR5, contributing to the elevation of white adipose tissue thermogenesis [34]. Bloom of *Akkermansia muciniphila* and a reduction in *Lactobacillus* in high fat/high sucrose (HFHS)-fed mice result in an increase in α/β-murocholic acid (MCA), DCA, and UDCA, which activate TGR5 and increase energy expenditure [35]. *Akkermansia muciniphila is* significantly decreased in lean individuals with T2D than their counterparts, which also has a negative correlation with serum 3β-CDCA levels and a positive correlation with insulin secretion and FGF15/19 concentrations [36].

#### 4.2.2. The Molecular Mechanism of How TGR5 Influences NAFLD

GLP-1 is secreted by enteroendocrine L cells and subsequently potentiates postprandial insulin secretion by pancreatic β cells. TGR5-mediated GLP-1 secretion is dependent on the cAMP mechanism [44]. TGR5 is found to activate mTORC1 signaling in several studies [69,70] and TGR5-mTORC1 signaling in the ileum is also considered to regulate the secretion of GLP-1 [69]. However, FXR activation in L cells decreases the secretion of GLP-1 by interfering with the glucose-responsive factor ChREBP [71] and inhibiting SCFAs induced GLP-1 secretion by decreasing free fatty acid receptor 2 (FFAR2) expression [72]. TGR5 activation in primary adipocytes is necessary for the respiration increase induced by mitochondrial fission through activation of extracellular signal-regulated kinase (ERK)/dynamin-1-like protein (DRP1) signaling [73]. It is reported that TGR5 activation in macrophages reduces macrophage migration depending on mTORC1 [70], thus ameliorating liver inflammation in NAFLD. TGR5 activation in hepatocytes also negatively regulates the hepatic inflammatory response by suppressing the NF-κB pathway by the mediation of the interaction between IκBα and β-arrestin2 [74].

### 4.3. Other Bile Acid Receptors

Other members of the nuclear receptor family, including the pregnane X receptor/steroid and xenobiotic-sensing receptor (PXR/SXR, NR1I2), constitutive androstane receptor (CAR, NR1I3), and the vitamin D3 receptor (VDR, NR1I1), also play a role in transducing the impact on NAFLD caused by bile acids and gut microbiota.

VDR gene variation has been discovered to contribute to significant shifts in the microbiota in a study performing a genome-wide association study of the gut microbiota. The upregulation of VDR is accompanied by a much lower abundance of *Parabacteroides*, which is positively correlated with LCA concentration [75]. Interestingly, another research study reveals that sleeve gastrectomy leads to a decrease in *Clostridia*, resulting in a decrease in LCA in the intestine and an increase in the portal vein. LCA induces hepatic mSult2A1/hSULT2A expression to drive cholic acid-7-sulfate (CA7S) production via activating VDR. CA7S in turn induces GLP-1 secretion in intestinal L cells and improvement of diabetic phenotypes after sleeve gastrectomy in mice [37]. The complicated mechanism further illustrates the consistent interplay between gut microbiota and bile acids in metabolism regulation. However, the role of PXR is confused as the activation or knockdown of PXR both promote de novo lipogenesis and steatosis [76].

Other cell surface receptors belonging to the GPCR, including S1PR2, formyl-peptide receptors (FPRs), and muscarinic acetylcholine receptors (mAChRs) [68] also bind with bile acids and transfer regulatory information in NAFLD. S1PR2 is activated by TCA and other conjugated bile acids, but not unconjugated bile acids [77]. It is proved that activation of S1PR2 induces the proliferation of cholangiocytes and cholestasis-induced liver injury [78,79]. But S1PR2−/− mice rapidly develop overt fatty livers in a high-fat diet as compared with wild-type mice [80]. Activation of S1PR2 increases sphingosine kinase 2 (Sphk2) and sphingosine-1-phosphate (S1P), which induces genes involved in lipid and sterol metabolism and transportation in the liver [81]. Sphk2 facilitates upregulation of genes encoding enzymes in fatty acid transport and oxidation [82]. FPRs are expressed in neutrophils and monocytes, but CDCA and DCA could interfere with the binding of FPRs with its agonist N-formylmethionyl-leucyl-phenylalanine, thus decreasing the liver inflammation response [83,84].

## 5. The Therapeutic Methods Targeting Microbiota–Bile Acid Pathway

### 5.1. The Clinical Application of Microbiota in NAFLD Treatment

Considering the significant effect of the gut microbiota on NAFLD, fecal microbiota transplantation (FMT) has been tested in patients with metabolic syndrome and obesity. Although the improvement of insulin sensitivity is reported, mild alteration of other metabolic parameters is observed [85]. However, specific probiotic treatments show satisfactory clinical efficacy. The mixture of eight probiotic strains (*Streptococcus thermophilus*, *bifidobacteria* [*B. breve*, *B. infantis*, *B. longum*], *Lactobacillus acidophilus*, *L. plantarum*, *L. paracasei*, and *L. delbrueckii* subsp. *bulgaricus*) decreases the BMI of obese children with biopsy-proven NAFLD and increases the level of GLP-1 [86]. *Lactobacillus rhamnosus strain GG* treatment reveals a significant decrease in alanine aminotransferase in children with obesity-related liver disease [87]. Another research study using similar bacteria including *Streptococcus thermophilus*, *Bifidobacterium breve*, *Bifidobacterium longum*, *Lactobacillus acidophilus*, *Lactobacillus bulgaricus*, *Lactobacillus rhamnosus*, and *Lactobacilluscasei* combined with lifestyle modification reports the amelioration of liver inflammation in patients with NAFLD [88]. The beneficial effect of *Lactobacilli*, *Bifidobacteria*, and *Streptococcus thermophilus* on NAFLD has been validated by various clinical research studies [86,88,89,90,91,92]. Moreover, the application of bacteriophages (phages) targeting specific harmful bacteria in NAFLD is a new potential treatment approach. The success of administrating phages against cytolytic *E. faecalis* to reduce the severity of ethanol-induced liver disease in the mice provides the first evidence that strategies to target single gut microbes might be developed for treatment of NAFLD [93].

### 5.2. The Clinical Application of Bile Acid or Bile Acid Receptor Agonists in NAFLD Treatment

Therapies using natural bile acids, such as UDCA, have been proven to be successful in the therapy for PBC (primary biliary cirrhosis). However, it has only limited therapeutic efficacy in NASH in several randomized controlled studies [94,95]. The UDCA-homolog norursodeoxycholic acid (norUDCA) seems to be effective for NAFLD treatment with a significant reduction in serum ALT in the clinical trial [96]. Aramchol is a novel synthetic lipid molecule obtained by conjugating two natural components, CA (bile acid) and arachidic acid (saturated fatty acid), through a stable amide bond. It could significantly inhibit the activity of a gene involved in lipid synthesis named stearoyl coenzyme A desaturase 1 (SCD1). The clinical trial confirmed its positive effect on the reduction in liver fat content in NAFLD patients [97].

The impairment of intestinal bile acid absorption by inhibiting ASBT improves features and insulin sensitivity of NAFLD in HFD-fed mice [98]. In phase 1 studies, the ABST inhibitor volixibat could decrease the level of total and low-density lipoprotein (LDL) cholesterol [99]. However, in the double-blind, phase II study, volixibat had no therapeutic impact on steatosis or liver injury in NASH [100]. Further studies on ASBT inhibitors need to be performed to further assess the efficacy and safety of this approach in humans.

FXR-specific activator OCA (6α-ethylCDCA) is proven to lead to weight loss and improve histological changes in clinical trials of NASH [101,102]. Other FXR agonists such as cilofexor, tropifexor, and MET409 could reduce hepatic fat content in NASH patients [103,104,105]. However, OCA therapy is associated with an increase in VLDL and LDL particles and a reduction in HDL [106], which may originate from the ability of FXR activation to blunt bile acid synthesis and LDL clearance via repression of CYP7A1. Moreover, FXR activation upregulates the expression of flavin mono-oxygenase3 (FMO3), which oxidized the microbial metabolite of choline and carnitine to trimethylamineN-oxide (TMAO) to contribute to the development of atherosclerosis [107]. So rigorous measurements of the side effects on the cardiovascular system should be taken when using FXR agonists. Considering the beneficial effect of FGF15/19, the FGF19 analog is created and tested for the treatment of NAFLD. Although aberrant FGF19-FGFR4 signaling has been identified in hepatocellular carcinoma [108], using non-tumorigenic FGF19 analog NGM282 produces rapid and significant reductions in liver fat content in NASH patients in the phase II clinical trial without promoting carcinogenesis [109].

There is limited clinical evidence about the effect of the TGR5 activator on the treatment of NAFLD. TGR5 agonist SB-756050 has been tested in T2D patients, but it shows highly variable pharmacodynamic effects in the subjects. It might be explained by the short duration of exposure (6 days) and the small number of subjects (51 patients) [110]. Notably, TGR5 agonists might be toxic to cardiomyocytes [111] and it is proved by a study in which TGR5 agonists result in reflex tachycardia in dogs [112]. TGR5 agonists also increase gallbladder volume in mice [113]. However, there is also an experiment proving the beneficial effect of TGR5 agonists on the cardiovascular system [114]. Larger-scale clinical trials are needed to evaluate the effect of TGR5 agonists in NAFLD treatment. Until now, other bile acid-stimulating receptors such as PXR, CAR, and VDR have not been subjected to anti-NAFLD agent development [115].

## 6. Conclusions and Perspectives

The interaction between gut microbiota and bile acids in entero-hepatic tissues regulates the metabolism of NAFLD patients. On one hand, gut microbiota dysbiosis in NAFLD catalyzes the transformation of bile acids depending on BSH, 7-dehydroxylation, HSDH, or the amide conjugation reaction. Consequently, the alteration of bile acids exerts an influence on NAFLD via binding with bile acid receptors that are distributed in different sites, thus regulating the lipid or glucose metabolism signaling. On the other hand, a change in the bile acid pool causes the composition shift in gut flora in reverse. As bile acids act as anti-microbial agents by damaging bacterial membranes and altering intracellular macromolecular structures through detergent actions, only microbial populations able to tolerate high bile acid concentrations can survive in the gut [6], which lays the foundation for the transformation of intestinal microbiota caused by bile acids. Altered gut microbiota will have an influence on the NAFLD development by managing the microbial metabolites such as lipopolysaccharide, SCFAs, and other substances. We summarize the gut microbiota and bile acid alteration in NAFLD: for example, *Clostridium XlVa* (*OTU57*) has a positive correlation with DCA and downregulates hepatic gluconeogenesis through activating FXR-SHP-FOXO1 signaling, and the bloom of *Akkermansia muciniphila* with the increase in α/β-MCA, DCA, and UDCA activates TGR5 and accelerates energy expenditure. However, the upper hand in driving the pathogenesis of NAFLD is difficult to determine given the complex interplay between gut microbiota and bile acids.

As gut microbes are a very important ecosystem in NAFLD patients, strategies to use beneficial gut microbiota in an additive way have been used as a therapeutic approach. FMT has been investigated accordingly. Some research studies report the improvement of symptoms in patients with metabolic syndrome [116,117]; however, other trials observe mild and no significant changes in metabolic parameters [118,119]. Importantly, the administration of probiotics or synbiotics to patients with NAFLD significantly reduces the levels of alanine aminotransferase, liver stiffness measurements, and hepatic steatosis [120]. However, current research is still limited to a few known types of bacteria, and it is necessary to broaden the scope of research and explore other possible species to further complete the therapy strategies for NAFLD patients.

The encouraging clinical results of using OCA or other FXR agonists in ameliorating hepatic steatosis have demonstrated the efficiency of bile acid receptor agonists in the treatment of NAFLD. It is confirmed that FXR activation in the liver shows improvement in liver metabolic disorders. However, the contradictory results in hepatic and intestine FXR activation in the NAFLD model warrant the design of the specific liver FXR agonists. Although TGR5 activation is proven to increase energy expenditure and improve glucose metabolism, the side effects on the cardiovascular system and gall bladder should be considered in the future development of TGR5 agonists.

## Figures and Tables

**Table 1:** Regulation of bile acid metabolism by intestinal flora.

Microbe	Bile Acid	Mechanism of Regulating Bile Acid Metabolism	Reference
*Firmicutes*, *Bacteroidetes*, and *Actinobacteria*	deconjugation	BSH	[9]
*Clostridium*	CDCA→LCA CA→DCA	7-dehydroxylation	[9]
*Clostridium absonum*	CDCA→UDCA	7-α/β-isomerization	[10]
*Actinobacteria*, *Proteobacteria*, *Firmicutes*, and *Bacteroidetes*	oxidation and epimerization	HSDH	[11]
*Clostridia bolteae* strain WAL-14578 and strain CC43001B	Phe-chol, Tyr-chol, and Leu-chol	amide conjugation	[12]

**Table 2:** The alteration of gut microbiota and bile acids in metabolism disorder.

Microbe	Bile Acid	Mechanism of Influencing NAFLD	Outcome	Reference
*Bacteroidetes*, *Lachnospiraceae*, *Ruminococcaceae*	GDCA, 7-ketodeoxycholic acid, dehydrocholic acid	undefined	increasing with NAFLD activity and fibrosis stage in human	[18]
*Parabacteroides distasonis*	non-12α-hydroxylated bile acids, LCA and UDCA	increased thermogenesis	ameliorating weight regain in mice	[19]
*Firmicutes* and *Proteobacteria*	reduction in secondary bile acids	undefined	aggravating insulin resistance in human	[20]
*Ruminococcaceae_NK4A214_group*	CA, LCA and TUDCA	undefined	male infertility in metabolic syndrome sheep model	[21]
*Barnesiella*, *Clostridium XlVa*	TCA, TCDCA, CA and CDCA	activating FXR-SHP -FOXO1 signaling	preventing the development of metabolic disorders in mice	[22]
*Bifidobacterium*, *Bacteroides vulgatus*	the primary bile acids	undefined	treatment of children with obesity	[24]
*Veillonella*	GCA, GCDCA, GDCA, DCA	SCFAs	enhancing NASH patients’ performance	[15]
*Desulfovibrionales*	enhanced cecal secondary bile acids	generation of H_2_S	cholesterol gallstone disease	[25]
*Clostridium*	suppress secondary bile acid	hepatic FXR activation	ameliorating NASH in mice	[26]
*Ruminococcus*, *Oscillospira*, *Sutterella*, *Allobaculum*	CA, T-βMCA	hepatic FXR inhibition	contributing to hepatic insulin resistance and inflammation in mice	[27]
BSH-enriched bacteria	conjugated bile acids	inhibiting the intestinal FXR-FGF15 signaling pathway	reducing hepatic cholesterol in mice	[28]
*Bacteroides fragilis*	GUDCA	inhibiting the intestinal FXR	improving metabolic dysfunction both in humans and mice	[29]
*Firmicutes*, *Bacteroides*	DCA, LCA	FXR activation in the myeloid cells leading to production and activation of type I IFN	intensifying gut inflammation in mice	[30]
*Lactobacillus rhamnosus GG*	T-βMCA	activating the intestinal FXR	amelioration of liver inflammation and fibrosis in mice	[31]
*Acetatifactor* and *Bacteroides*	LCA	activating TGR5 to stimulate GLP-1 secretion	improving metabolism indicators in mice	[32]
*Lachnospiraceae* and *Eggerthellaceae*	HDCA	activating TGR5 to stimulate GLP-1 secretion	improving glucose metabolism in mice	[33]
*Bacteroides vulgatus* and *Ruminococcus torques*	TCA, DCA	activating TGR5	elevation of white adipose tissue thermogenesis in mice	[34]
*Akkermansia muciniphila*, *Lactobacillus*	α/β-MCA, DCA, and UDCA	activating TGR5	increasing energy expenditure in mice	[35]
*Akkermansia muciniphila*	3β-CDCA	FGF15/19	protecting against glucose intolerance in mice	[36]
*Clostridia*	LCA	LCA-VDR-SULT2A-CA7S-GLP-1 signaling	the improvement of diabetic phenotypes in mice sleeve gastrectomy	[37]

## Data Availability

Not applicable.

## Abbreviations

ABCG5/8	ATP-binding cassette sub-family G member 5/8
apo	apolipoprotein
ASBT	apical sodium-dependent bile acid transporter
BaiCD	bile acid operon
BSH	bile salt hydrolase
CA	cholic acid
CA7S	cholic acid-7-sulfate
CAR	constitutive androstane receptor
CDCA	chenodeoxycholic acid
ChREBP	carbohydrate-responsive element binding protein
CREB	cAMP regulatory element-binding protein
CYP7A1	cholesterol 7-alpha- hydroxylase
DCA	deoxycholic acid
DHA	docosahexaenoic acid
DRP1	dynamin-1-like protein
EPA	eicosapentaenoic acid
ERK	extracellular signal-regulated kinase
FEX	fexaramine
FGF	fibroblast growth factor
FMO3	flavin mono-oxygenase3
FMT	fecal microbiota transplantation
FPRs	formyl-peptide receptors
FXR	farnesoid X Receptor
G6Pase	glucose-6-phosphatase
GCA	glycocholic acid
GCDCA	glycochenodeoxycholic acid
GDCA	glycodeoxycholate
GPCR	G-protein-coupled receptors
GUDCA	glycoursodeoxycholic acid
HFD	high-fat diet
HFHS	high fat/high sucrose
HIF-2α	hypoxia-inducible factor 2α
HNF4α	hepatocyte nuclear factor 4α
HSCs	hepatic stellate cell
HSDH	hydroxysteroid dehydrogenase
LCA	lithocholic acid
LDL	low-density lipoprotein
Leu-chol	leucocholic acid
L-PK	liver-type pyruvate kinase gene
mAChRs	muscarinic acetylcholine receptors
MCA	murocholic acid
MTP	microsomal triglyceride transfer protein
NAFLD	nonalcoholic fatty liver disease
NASH	nonalcoholic steatohepatitis
norUDCA	norursodeoxycholic acid
OCA	obeticholic acid
OSTα/β	organic solute transporter-alpha and -beta
PBC	primary biliary cirrhosis
PGC-1α	peroxisome proliferator-activated receptor γ coactivator-1α
Phe-chol	phenylalanocholic acid
PPAR γ	peroxisomal proliferator-activated receptor γ
PXR	pregnane X receptor
PXR/SXR	pregnane X receptor/steroid and xenobiotic-sensing receptor
RYGB	Roux-en-Y gastric bypass
S1PR	sphingosine-1-phosphate receptor
S1PR2	sphingosine 1-phosphate receptor 2
SCFAs	short-chain fatty acids
SHP	small heterodimer partner
SIRT	sirtuin
Sphk2	sphingosine kinase 2
SREBP-1c	sterol regulatory binding protein-1c
TCA	taurine-conjugated cholic acid
TCDCA	taurochenodeoxycholic acid
TGR5	takeda G protein-coupled receptor 5
TLCA	taurolithocholic acid
TMAO	trimethylamineN-oxide
TUDCA	tauroursodeoxycholic acid
Tyr-chol	tyrosocholic acid
T-α/βMCA	taurine-α/β-muricholic acid
UDCA	ursodeoxycholic acid
VDR	vitamin D receptor
